# Daily Injection of the β2 Adrenergic Agonist Clenbuterol Improved Muscle Glucose Metabolism, Glucose-Stimulated Insulin Secretion, and Hyperlipidemia in Juvenile Lambs Following Heat-Stress-Induced Intrauterine Growth Restriction

**DOI:** 10.3390/metabo14030156

**Published:** 2024-03-07

**Authors:** Rachel L. Gibbs, James A. Wilson, Rebecca M. Swanson, Joslyn K. Beard, Zena M. Hicks, Haley N. Beer, Eileen S. Marks-Nelson, Ty B. Schmidt, Jessica L. Petersen, Dustin T. Yates

**Affiliations:** 1 Department of Animal Science, University of Nebraska-Lincoln, Lincoln, NE 68583, USA; rachel.gibbs@huskers.unl.edu (R.L.G.);; 2 Department of Biology, University of Nebraska-Omaha, Omaha, NE 68182, USA; jameswilson@unomaha.edu

**Keywords:** carbohydrate metabolism, developmental origins of health and disease, DOHaD, fetal programming, metabolic dysfunction

## Abstract

Stress-induced fetal programming diminishes β2 adrenergic tone, which coincides with intrauterine growth restriction (**IUGR**) and lifelong metabolic dysfunction. We determined if stimulating β2 adrenergic activity in IUGR-born lambs would improve metabolic outcomes. IUGR lambs that received daily injections of saline or the β2 agonist clenbuterol from birth to 60 days were compared with controls from pair-fed thermoneutral pregnancies. As juveniles, IUGR lambs exhibited systemic inflammation and robust metabolic dysfunction, including greater (*p* < 0.05) circulating TNFα, IL-6, and non-esterified fatty acids, increased (*p* < 0.05) intramuscular glycogen, reduced (*p* < 0.05) circulating IGF-1, hindlimb blood flow, glucose-stimulated insulin secretion, and muscle glucose oxidation. Daily clenbuterol fully recovered (*p* < 0.05) circulating TNFα, IL-6, and non-esterified fatty acids, hindlimb blood flow, muscle glucose oxidation, and intramuscular glycogen. Glucose-stimulated insulin secretion was partially recovered (*p* < 0.05) in clenbuterol-treated IUGR lambs, but circulating IGF-1 was not improved. Circulating triglycerides and HDL cholesterol were elevated (*p* < 0.05) in clenbuterol-treated IUGR lambs, despite being normal in untreated IUGR lambs. We conclude that deficient β2 adrenergic regulation is a primary mechanism for several components of metabolic dysfunction in IUGR-born offspring and thus represents a potential therapeutic target for improving metabolic outcomes. Moreover, benefits from the β2 agonist were likely complemented by its suppression of IUGR-associated inflammation.

## 1. Introduction

Skeletal muscle is a target tissue for stress-induced thrifty fetal programming, and its disproportional impairment relative to brain and skeletal growth leads to the hallmark asymmetric intrauterine growth restriction (**IUGR**) of the chronically stressed fetus [1,2]. Programmed changes in IUGR fetal muscle contribute to metabolic adaptations that manifest in late gestation [3,4] and predispose afflicted individuals to lifelong metabolic dysfunction [5]. In livestock, this metabolic programming causes IUGR-born offspring to exhibit reduced growth efficiency and poor body composition that diminishes carcass merit [6]. Adaptive fetal programming is primarily a response to chronic intrauterine hypoxia and malnutrition brought on by placental insufficiency [7,8]. The sustained hypercatecholaminemic response of the fetus helps to preserve its limited nutrients by redirecting utilization away from skeletal muscle and adipose tissues [9,10,11]. Over time, however, fetal tissues adapt to chronic catecholamine exposure by reducing responsiveness to adrenergic stimulation [12,13,14]. In skeletal muscle and myoblasts (i.e., muscle stem cells), β2 adrenoceptors comprise about 90% of the adrenergic receptor populations [15,16], and their reduction is the primary mechanism of desensitization. Reduced skeletal muscle β2 adrenoreceptor content persists in IUGR-born offspring and coincides with diminished muscle mass and oxidative metabolism [2,14,17]. Abhorrent adrenergic regulation of IUGR pancreatic islets also disrupts insulin production and secretion, although this is primarily due to α adrenergic changes [13,18,19,20]. Under normal conditions, β2 adrenergic pathways stimulate muscle growth and metabolic efficiency [21,22] by increasing mitochondrial number, activating the Akt and mTORC pathways, and stimulating IGF-1 production [23,24]. They also temper inflammation, which is elevated by IUGR conditions [25,26]. Fetal studies indicate that adrenergic adaptations may explain as much as half of the IUGR metabolic phenotype [18,19]. We hypothesized that this remained the case after birth and that diminished β2 adrenergic tone would be an effective target for improving postnatal muscle metabolism and glucose homeostasis following heat stress-induced IUGR. Our objective was to test this hypothesis by determining whether daily intramuscular injection of the β2 adrenergic agonist, clenbuterol HCl, would improve skeletal muscle glucose metabolism, pancreatic β cell function, and lipid homeostasis in IUGR juvenile lambs.

## 2. Materials and Methods

### 2.1. Animals and Experimental Design

The experiments described in this manuscript were approved by the Institutional Animal Care and Use Committee at the University of Nebraska–Lincoln (protocols 2381 and 2155), which is accredited by AAALAC International. The lambs used for this study were previously described in detail [17]. To generate IUGR lambs, Polypay-crossbred ewes were timed-mated to a single ram, fed ad libitum, and housed under heat-stress conditions (35 °C × 12 h/day, 40 °C × 12 h/day, 35% relative humidity, temperature–humidity index = 84 to 86) from the 40th to the 95th day of gestation and under thermoneutral conditions for the remainder of gestation. Lambs were weaned at birth, fed pooled colostrum for the first 36 h, and reared on commercial formula (Land O’Lakes, Arden Hills, MN, USA) until 30 days after birth. From days 30 to 45, lambs were transitioned to a full grain diet, which was fed exclusively from days 45 to 60. From birth, IUGR-born lambs received IM injections of 800 ng/kg of clenbuterol HCl (**IUGR+CLEN**; n = 11 lambs born to 7 ewes; 2 singletons, 9 twins; 6 males and 5 females) or saline carrier each day (**IUGR**; n = 12 lambs from 8 ewes; 2 singletons and 10 twins; 5 males and 6 females). Controls (n = 13 lambs born to 8 ewes; 2 singletons, 11 twins; 7 males and 6 females) were born to thermoneutral ewes that were pair-fed to the average of the heat-stressed animals. These lambs also received daily saline injections. Indirect calorimetry was performed on days 30 and 58 to assess whole-body oximetry. Surgical hindlimb vascular catheterizations were performed on day 54, and daily blood samples were collected from days 56 to 60. Square-wave hyperglycemic clamp studies were performed on day 57 to assess β cell function. Hyperinsulinemic–euglycemic clamp studies were performed on day 58 to assess hindlimb glucose metabolism. Lambs were euthanized via barbiturate overdose on day 60 for necropsy.

### 2.2. Surgical Catheterization

Surgical hindlimb preparations were performed under general anesthesia to place indwelling femoral catheters for blood sampling and substrate infusions, as previously described [14,27]. Briefly, gas-sterilized Tygon catheters were placed with the tip advanced into the external iliac artery via the femoral artery for arterial blood sampling. A second set of catheters was placed in the common iliac vein via the femoral vein for substrate infusion, and a third set was placed in the contralateral common iliac vein for venous blood sampling. A Precision S-series perivascular blood flow probe (Transonic Systems, Inc., Ithaca, NY, USA) was placed around the contralateral femoral artery to estimate blood flow rate through the hindlimb. The descending lateral circumflex artery on this hindlimb was ligated and severed. The probe cable and catheters were tunneled subcutaneously, exteriorized, and stored in mesh pouches sutured to the skin. All catheters were flushed with heparinized saline twice daily.

### 2.3. In Vivo Metabolic Analyses

#### 2.3.1. Glucose-Stimulated Insulin Secretion

A square-wave hyperglycemic clamp was performed to measure basal (i.e., under resting conditions) and 2nd-phase glucose-stimulated insulin secretion as previously described [14,27]. Briefly, a series of three arterial blood samples were collected in 5-min intervals to measure basal blood glucose and plasma insulin concentrations. Afterward, an intravenous bolus of 33% dextrose (Vet One; Boise, ID, USA) in saline was administered to deliver 250 mg/kg glucose. The bolus was followed immediately by a continuous, variable-rate 33% dextrose infusion to reach a target steady-state hyperglycemic condition of 2.5-fold basal glucose concentration (±10%). After a minimum of 20 min from the dextrose bolus, another three arterial blood samples were collected during steady-state hyperglycemia in 5-min intervals. Due to catheter failures in some lambs, hyperglycemic clamp studies were performed on 13 controls, 11 IUGR lambs, and 10 IUGR+CLEN lambs.

#### 2.3.2. Hindlimb Glucose Metabolism

Hyperinsulinemic–euglycemic clamps (**HECs**) were performed to assess hindlimb-specific glucose metabolism under basal and insulin-stimulated conditions as previously described [14,27]. Briefly, lambs were infused with [^14^C(U)]-D-glucose (37.2 μCi/mL; Perkin-Elmer Inc., Waltham, MA, USA) at 2 mL/h. After 40 min, basal arterial and venous blood samples were simultaneously collected in 5-min intervals (4 total pairs). Hyperinsulinemia was then induced by infusing insulin (250 mU/kg; Humulin-R; Lilly, Indianapolis, IN, USA) at 4 mU/kg/h. To maintain concurrent euglycemia (basal blood glucose ± 10%), intravenous 33% dextrose was bolused and infused at a variable rate. After 1 h, 4 additional pairs of simultaneous arterial and venous blood samples were collected in 5-min intervals under HEC conditions. Blood flow rates into the hindlimb were averaged for basal and HEC periods. Hindlimb-specific glucose utilization and glucose oxidation rates were estimated from arteriovenous differences in the concentrations of glucose and ^14^CO_2_, respectively. Differences were normalized to average blood flow rates and to hindlimb weight at necropsy. Due to catheter and/or blood flow probe failures in some lambs, HEC studies were performed on 11 controls, 10 IUGR lambs, and 8 IUGR+CLEN lambs.

### 2.4. Blood Hormones and Metabolites

Whole blood samples were analyzed for gases, metabolites, and hormones, as previously described [14,27]. Briefly, glucose, lactate, pH, partial pressure of CO_2_ (**pCO_2_**), partial pressure of O_2_ (**pO_2_**), HCO_3_^−^, base excess, oxyhemoglobin, carboxyhemoglobin, Na^+^, K^+^, Cl^−^, and Ca^2+^ were measured in heparinized whole blood with an ABL90 FLEX blood gas analyzer (Radiometer, Brea, CA, USA). Plasma was separated from EDTA-treated whole blood via centrifuge (14,000× *g*, 5 min), and commercial ELISA kits were used to determine plasma concentrations of insulin (ALPCO, Salem, NH, USA), insulin-like growth factor-1 (**IGF-1**; ALPCO), tumor necrosis factor-alpha (**TNFα**; Wuhan Fine Biotech Co., Wuhan, Hubei, China), interleukin-6 (**IL-6**; Biomatik, Kitchener, ON, Canada), and non-esterified fatty acids (**NEFAs**; Fujifilm, Richmond, VA, USA). Intra-assay and inter-assay coefficients of variance were calculated for all ELISAs and were not more than 15%. Blood plasma urea nitrogen (**BUN**) concentrations, total triglyceride concentrations, and high-density lipoprotein (**HDL**)-bound cholesterol concentrations were measured on a Vitros 250 Analyzer (Ortho Diagnostics, Raritan, NJ, USA). These tests were performed by the Biomedical and Obesity Research Core at the University of Nebraska. Total cholesterol concentrations were assessed but were generally below detectability.

### 2.5. Whole-Body Oxidative Metabolism

To estimate whole-body oxidative metabolism, O_2_ consumption rates and CO_2_ production rates were determined by indirect calorimetry as previously described [28,29], with some modifications. Lambs were placed into Panepinto slings, and a clear-plastic globe sealed with a surgical nose cone gasket was secured over the head of the lamb, as shown in Supplementary Figure S1. Atmospheric air was pumped into the globe through an inlet hose at a constant rate of 110 L/min. The O_2_ and CO_2_ content of the air leaving the globe through an outlet hose was measured with a Fox Box Respirometry System (Sable Systems, Las Vegas, NV, USA) that was calibrated with a standard of 1% CO_2_ with N_2_ as the balance gas. O_2_ was calibrated with atmospheric gas (20.95% O_2_). Measurements were obtained over two 30 min periods, each of which followed a 5-min baseline reading of atmospheric air. Values for O_2_ consumption rates, CO_2_ production rates, and metabolic rates (J/h) were averaged between the two periods.

### 2.6. Ex Vivo Skeletal Muscle Glucose Metabolism

Both *flexor digitorum superficialis* (**FSD**) muscles were collected at necropsy and used to assess ex vivo glucose metabolism as previously described [14,27]. Briefly, muscles were split into longitudinal strips (106.5 ± 6.2 mg) that were pre-incubated for 1 h at 38 °C and 5% CO_2_ in Krebs–Henseleit Buffer (**KHB**) with 5 mM glucose (MilliporeSigma, St. Louis, MO, USA). Incubations included no additional additive (basal), 5 mU/mL insulin (Humulin-R), or 10 ng/mL TNFα (MilliporeSigma). Following pre-incubation, muscle strips were washed in glucose-free KHB with the respective additive for 20 min. To measure glucose oxidation, muscle strips (3 technical replicates/condition) were placed in one side of sealed dual-well chambers and incubated in KHB media gassed with 95% O_2_ and containing the respective additive and 5 mM [^14^C(U)]-D-glucose (0.25 μCi/mmol) for 2 h. The adjoining well of each chamber contained 1 M NaOH (MilliporeSigma). After 2 h incubations were complete, chambers were cooled on ice, and the wells containing KHB were injected with 2 M HCl (MilliporeSigma) and allowed to incubate at 4 °C for an additional 2 h. This allowed bicarbonate-bound ^14^CO_2_ to be released from the media and captured by the NaOH in the adjoining well. The NaOH was collected and combined with UltimaGold scintillation fluid (Perkin-Elmer), and ^14^CO_2_ concentrations were calculated from ^14^C-specific activity determined by liquid scintillation with a Beckman-Coulter 1900 TA LC counter (Beckman-Coulter, Fullterton, CA, USA). To measure glucose uptake, muscle strips were incubated in KHB media containing the respective additive and 1 mM [^3^H]2-deoxyglucose (300 μCi/mmol; Perkin-Elmer) and 39 mM ^14^C-mannose (1.25 μCi/mmol; Perkin-Elmer) for 20 min. After the 20 min incubation, muscle strips were cooled on ice, washed in cold phosphate-buffered saline (**PBS**), and lysed in 2 M NaOH. The concentration of [^3^H]2-deoxyglucose for each lysate was then calculated from ^3^H-specific activity determined by liquid scintillation. The volume of extracellular fluid in the muscle lysate was calculated from ^14^C-specific activity, and the extracellular fluid contribution to ^3^H-specific activity was subtracted from the lysate total. Ex vivo glucose uptake and oxidation rates were normalized to the time in incubation and muscle strip mass.

### 2.7. Pancreatic Islet Morphometrics

Pancreatic samples were collected at necropsy and stained for β cell and α cell populations as previously described [30]. Briefly, pancreatic tissues were fixed with 4% paraformaldehyde in PBS, embedded in Tissue-Tek OCT compound (Fisher Scientific, Waltham, MA, USA), and stored at −80 °C. Cryosections were prepared at 8 µm and mounted on charged microscope slides (Fisher Scientific). These were dried for 30 min at 37 °C and rehydrated with PBS. For antigen retrieval, slides were first boiled (4 × 5 min) and then cooled (2.5 h at room temperature) in 10 mM citric acid. To block non-specific staining, slides were incubated in 0.5% NEN blocking buffer (Perkin-Elmer) in PBS for 1 h in a humidifier at room temperature. Slides were then incubated overnight at 4 °C with primary antibodies diluted in PBS + 1% bovine serum albumin (MilliporeSigma). Negative controls were incubated in PBS + 1% bovine serum albumin without primary antibodies. Pancreas sections were stained with mouse antiserum raised against glucagon (1:500; clone K79bB10, MilliporeSigma) and counterstained with guinea pig antiserum raised against insulin (1:1000; Agilent Technologies, Inc., Santa Clara, CA, USA). Immunocomplexes were detected with affinity-purified immunoglobulin antiserum conjugated to Alexa Fluor 488 and Alexa Fluor 594 (1:500; Cell Signaling Technologies, Danvers, MA, USA). Immunofluorescent images were visualized on an Olympus IX73 and digitally captured with a DP80 microscope camera (Olympus, Center Valley, PA, USA). Images were analyzed with Olympus cellsSens Dimension software to determine glucagon and insulin areas within 20 islets/animal. Animals and experimental groups were de-identified prior to analysis.

### 2.8. Skeletal Muscle Protein Expression

Total protein was isolated from *semitendinosus*, *biceps femoris*, and *longissimus dorsi* muscles that had been snap-frozen at necropsy with liquid nitrogen. Protein isolates were used to determine protein content for TNFα receptor 1 (**TNFR1**) and interleukin 6 receptor (**IL6R**) as previously described [14,27]. *Semitendinosus* protein isolates were also used to determine IκBα content and relative myosin heavy chain (**MyHC**) content, as previously described [31]. Briefly, muscle samples were homogenized via sonication (3 × 5 s) in a low-salt TRIS-NaCl buffer + 2.5% protease + 2.5% phosphatase inhibitor and then centrifuged (14,000× *g*, 5 min, 4 °C). Total protein concentration was quantified from the supernatant utilizing a Pierce BCA Assay Kit (Thermo Fisher, Waltham, MA, USA). To assess MyHC proportions, a 40 μg protein aliquot was mixed with Bio-Rad 4× Laemmli sample buffer and heated at 70 °C for 10 min. Electrophoresis was performed with an upper running buffer containing 100 mM Tris, 150 mM glycine, 0.1% SDS, and 0.07% β-mercaptoethanol in distilled water and a lower running buffer consisting of 50 mM Tris, 75 mM glycine, and 0.05% SDS in distilled water at room temperature for 3 h at a constant voltage of 110 V. Gels were then stained overnight in Gel-Code Blue (Thermo Fisher) at room temperature, washed in double-distilled water, and imaged using an Odyssey infrared imaging system (LI-COR Biosciences, Lincoln, NE, USA). Quantification of bands for MyHC-I, MyHC-IIA, and MyHC-IIx was completed by densitometry (Image Studio Lite Ver 5.0; LI-COR) to estimate fiber type ratios. For Western immunoblots, 50 μg protein aliquots were mixed with Bio-Rad 4× Laemmli sample buffer and heated (95 °C, 5 min). Samples were brought to room temperature and then separated by SDS–PAGE before being transferred to poly-vinylidene fluoride low-fluorescent membranes (Bio-Rad) for TNFR1 and IL6R or nitrocellulose membranes for IκBα. Membranes were washed with TBS-T prior to primary antibody incubation and incubated with Bio-Rad EveryBlot Blocking Buffer (10 min, room temperature). Membranes were incubated with rabbit anti-serum raised against TNFR1 (1:500, C25C1, Cell Signaling), rabbit anti-serum raised against IL6R (1:1000, clone EPR24322-143; Abcam), or mouse anti-serum raised against IκBα (1:200, H-4; Santa Cruz Biotechnology, Dallas, TX, USA; 4 °C, overnight). Finally, membranes were washed in TBS-T and incubated for 1 h at room temperature with goat anti-rabbit or goat anti-mouse IR800 IgG secondary anti-serum (LI-COR). Membranes were scanned using an Odyssey Infrared System, and protein bands were analyzed with Image StudioLite Software 5.2. Each protein of interest was normalized to the total protein.

### 2.9. Muscle Glycogen Content

Intramuscular glycogen content was quantified from snap-frozen samples of *semitendinosus*, *biceps femoris*, and *longissimus dorsi* muscles collected at necropsy as previously described [13]. Briefly, 100 mg of muscle was homogenized in double-distilled water via sonication for 15 s, heated at 95 °C for 5 min, and centrifuged for 5 min at 14,000× *g*. Glycogen content was quantified from duplicate 5 μL aliquots of the supernatant with a commercial ELISA kit (Glycogen Assay Kit, MilliporeSigma). The inter-assay and intra-assay coefficients of variance were less than 10%.

### 2.10. Statistical Analysis

Necropsy data were analyzed by ANOVA via the mixed procedure of SAS 9.4 (SAS Institute, Cary, NC, USA) to assess the fixed effects of the experimental group (i.e., controls, IUGR, and IUGR+CLEN), sex (i.e., male or female), and birth number (i.e., singleton or twin). Interactions with sex and birth number were not assessed due to limitations in power. Means separation was performed by Fisher’s LSD test. Daily and weekly blood components and in vivo metabolic outputs were analyzed via the mixed procedure of SAS. Repeated measures were used to analyze the effects of the experimental group, age/study period, and interaction, as well as sex and birth number. The prevalence of each sex and birth number category within each experimental group is reported in the methods. Placental anastomosis is rare in sheep [32], and experimental placental insufficiency was assumed to be applied to each fetus individually. Lamb was the individual experimental unit. Significant differences were declared when *p*-values were less than 0.05, and tendencies were declared when *p*-values were less than 0.10. All data are presented as means ± standard errors of the mean.

## 3. Results

### 3.1. Growth Parameters

Growth and body composition metrics for these lambs were published previously by Gibbs et al. [17]. Briefly, the bodyweights of IUGR lambs were reduced by 24% at birth, 18% on day 30, and 16% on day 60. The average daily gain between 0 and 60 days of age was also 13% lower for IUGR lambs. Bodyweights were not improved for IUGR+CLEN lambs compared to IUGR lambs, and average daily gain was intermediate between controls and IUGR lambs. At necropsy, loin eye areas and FDS muscles were 14% and 15% smaller, respectively, for IUGR lambs than for controls, but size was improved for both muscles in IUGR+CLEN lambs. *Semitendinosus* fat content and fat-to-protein ratios were 30% and 34% greater, respectively, and protein content was 3% lower for IUGR lambs, but all were recovered to normal for IUGR+CLEN lambs.

### 3.2. Daily Blood Hormone Concentrations

**Figure 1:**
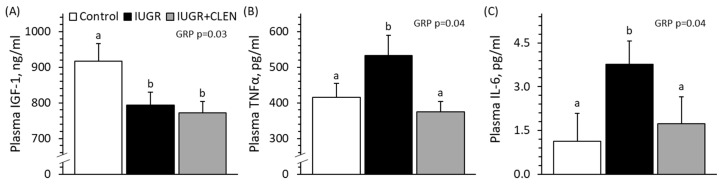
Daily blood hormone concentrations in clenbuterol-treated IUGR-born lambs. Arterial blood samples were assessed daily from days 56 to 60 in the controls (n = 13), untreated IUGR lambs (n = 11), and clenbuterol-treated IUGR lambs (IUGR+CLEN; n = 10). Data are presented for the mean plasma IGF-1 (**A**), TNFα (**B**), and IL-6 (**C**) concentrations. Effects of experimental group (GRP), day, and the interaction are noted where significant (*p* < 0.05). ^a,b^ Means with different superscripts differ (*p* < 0.05).

No experimental group × day interactions were observed for daily plasma IGF-1, TNFα, or IL-6 concentrations. Average daily plasma IGF-1 concentrations from days 56 to 60 were lower (*p* < 0.05) for IUGR and IUGR+CLEN lambs than for controls (Figure 1A). Average daily plasma TNFα and IL-6 concentrations from days 56 to 60 were greater (*p* < 0.05) for IUGR lambs but not IUGR+CLEN lambs compared to controls (Figure 1B,C). Plasma IGF-1 was greater (*p* < 0.05) for males than for females, and plasma IFG-1 and TNFα were greater (*p* < 0.05) for singletons than for twins. Plasma IL-6 was greater (*p* < 0.05) for twins than singleton lambs.

### 3.3. Glucose-Stimulated Insulin Secretion

**Figure 2:**
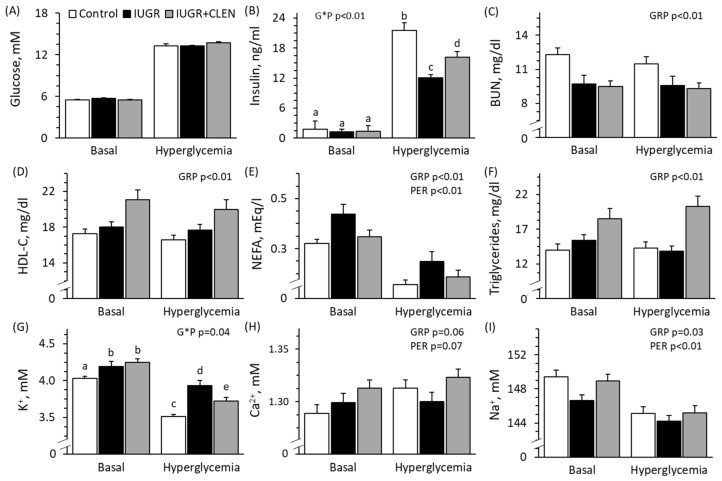
Glucose-stimulated insulin secretion in clenbuterol-treated IUGR-born lambs. Arterial blood samples were assessed under basal and steady-state hyperglycemia at 58 days of age in the controls (n = 13), untreated IUGR lambs (n = 11), and clenbuterol-treated IUGR lambs (IUGR+CLEN; n = 10). Data are presented for blood glucose (**A**), plasma insulin (**B**), blood plasma urea nitrogen (BUN) (**C**), HDL cholesterol (**D**), non-esterified fatty acid (NEFA) (**E**), triglyceride (**F**), K^+^ (**G**), Ca^2+^ (**H**), and Na^+^ (**I**) concentrations. Effects of the experimental group (GRP), period (PER), and the interaction (G*P) were evaluated and are noted where significant (*p* < 0.05). ^a,b,c,d,e^ Means with different superscripts differ (*p* < 0.05).

Experimental group × glycemic period interactions were observed (*p* < 0.05) for plasma insulin and blood K^+^ concentrations and tended to be observed (*p* = 0.06) for glucose-to-insulin ratios but not for any other blood components during the hyperglycemic clamp study. Blood glucose concentrations did not differ among groups and, by design, were increased (*p* < 0.05) 2.4-fold from basal during the hyperglycemic period for all groups (Figure 2A). Basal plasma insulin concentrations did not differ among groups, but glucose-stimulated insulin secretion was lower (*p* < 0.05) for IUGR lambs compared to controls and was intermediate for IUGR+CLEN lambs (Figure 2B). Glucose-to-insulin ratios were greater (*p* < 0.05) for IUGR (6.4 ± 1.9) and IUGR+CLEN (7.4 ± 2.2) lambs compared with controls (2.4 ± 0.2), regardless of period. Glucose-to-insulin ratios were also 84% lower (*p* < 0.05) during hyperglycemia than under basal conditions. Blood lactate concentrations were lower (*p* < 0.05) for IUGR lambs (0.59 ± 0.03 mM) but not for IUGR+CLEN lambs (0.75 ± 0.05 mM) than for controls (0.71 ± 0.03 mM), regardless of period. Blood lactate tended to be greater (*p* = 0.10) during hyperglycemia (0.72 ± 0.03 mM) than under basal conditions (0.65 ± 0.03 mM) for all groups. BUN concentrations were lower (*p* < 0.05) for IUGR and IUGR+CLEN lambs compared with controls, regardless of period (Figure 2C). Plasma HDL cholesterol was greater (*p* < 0.05) for IUGR and IUGR+CLEN lambs than for controls and greater (*p* < 0.05) for IUGR+CLEN lambs than for IUGR lambs, regardless of period (Figure 2D). Plasma NEFA concentrations were greater (*p* < 0.05) for IUGR lambs but not IUGR+CLEN lambs than for controls, regardless of period (Figure 2E). Plasma NEFA concentrations were lower (*p* < 0.05) during hyperglycemia than under basal conditions. Plasma triglyceride concentrations were greater (*p* < 0.05) for IUGR+CLEN lambs but not IUGR lambs compared to controls (Figure 2F). Basal blood K^+^ was greater (*p* < 0.05) for IUGR and IUGR+CLEN lambs than for controls (Figure 2G). Blood K^+^ during hyperglycemia was greater (*p* < 0.05) for IUGR lambs than for controls and was intermediate for IUGR+CLEN lambs. Blood Ca^2+^ tended to be greater (*p* = 0.06) for IUGR+CLEN lambs but not IUGR lambs compared with controls, regardless of period (Figure 2H), and tended to be greater (*p* = 0.07) during hyperglycemia than under basal conditions. Blood Na^+^ concentrations were lower (*p* < 0.05) for IUGR lambs but not IUGR+CLEN lambs than for controls, regardless of period (Figure 2I), and were lower (*p* < 0.05) during hyperglycemia than under basal conditions. Blood Cl^−^ was greater (*p* < 0.05) for IUGR+CLEN lambs (111.5 ± 0.8 mM) than for IUGR lambs (108.9 ± 0.5 mM), but neither group differed from controls (109.9 ± 0.6 mM). Blood Cl^−^ concentrations were also 2.4% lower (*p* < 0.05) during hyperglycemia than under basal conditions. Blood pH, hematocrit, and hemoglobin concentration were lower (*p* < 0.05) for IUGR+CLEN lambs but not IUGR lambs compared to controls, regardless of period (Supplementary Figure S2A–C). Hemoglobin concentration and hematocrit were also lower (*p* < 0.05) during hyperglycemia than under basal conditions. Base excess of the blood was greater (*p* < 0.05) for IUGR lambs and lower (*p* < 0.05) for IUGR+CLEN lambs than for controls, regardless of period (Supplementary Figure S2D). Oxyhemoglobin and pO_2_ were greater (*p* < 0.05) for IUGR and IUGR+CLEN lambs than for controls, regardless of period (Supplementary Figure S2E,F). Carboxyhemoglobin was greater (*p* < 0.05) for IUGR and IUGR+CLEN lambs than for controls and greater (*p* < 0.05) for IUGR+CLEN lambs than for IUGR lambs, regardless of period (Supplementary Figure S2G). Blood HCO_3_^−^ (26.3 ± 0.2 mM) and pCO_2_ (39.4 ± 0.3 mmHg) were not different among groups or between glycemic periods. Plasma insulin, blood pH, hemoglobin, and oxyhemoglobin were lower (*p* < 0.05), and pCO_2_, BUN, and NEFAs were greater (*p* < 0.05) for males than for females. Blood pH, pCO_2_, hemoglobin, hematocrit, oxyhemoglobin, K^+^, Cl^−^, Ca^2+^, and pO_2_ were greater (*p* < 0.05), and blood HCO_3_^−^, base excess, BUN, triglycerides, and NEFAs were lower (*p* < 0.05) for twins than singletons.

### 3.4. Insulin-Stimulated Hindlimb Metabolism

**Figure 3:**
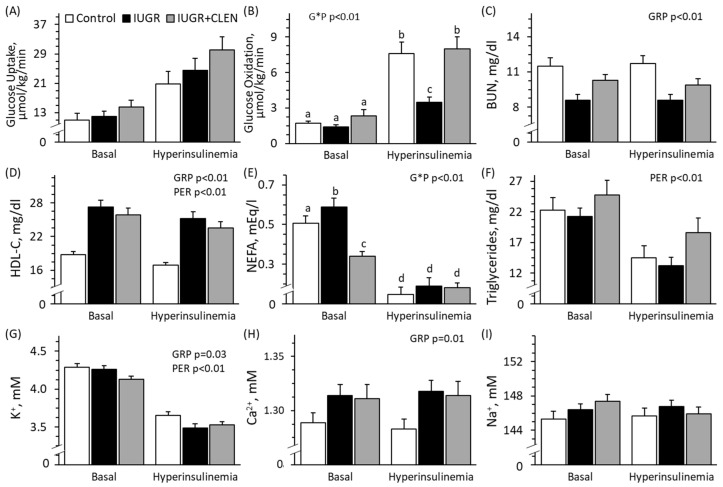
Metabolic indicators in clenbuterol-treated IUGR-born lambs. Hyperinsulinemic–euglycemic clamps were performed at 59 days of age in controls (n = 11), untreated IUGR lambs (n = 10), and clenbuterol-treated IUGR lambs (IUGR+CLEN; n = 10). Data are presented for hindlimb glucose uptake (**A**) and oxidation (**B**) rates, as well as blood plasma urea nitrogen (BUN) (**C**), HDL cholesterol (**D**), non-esterified fatty acid (NEFA) (**E**), triglyceride (**F**), K^+^ (**G**), Ca^2+^ (**H**), and Na^+^ (**I**) concentrations. Effects of the experimental group (GRP), period (PER), and the interaction (G*P) were evaluated and are noted where significant (*p* < 0.05). ^a,b,c,d^ Means with different superscripts differ (*p* < 0.05).

Experimental group × study period interactions were observed (*p* < 0.05) for hindlimb glucose oxidation rates and plasma NEFA concentrations but not for any other parameters assessed during the HEC study. Femoral blood flow rates were lower (*p* < 0.05) for IUGR lambs (75.5 ± 7.0 mL/min) but not for IUGR+CLEN lambs (97.5 ± 4.1 mL/min) than for controls (92.3 ± 9.7 mL/min), regardless of period. Hindlimb glucose uptake rates did not differ among experimental groups and increased (*p* < 0.05) 2-fold from basal during the HEC period for all groups (Figure 3A). Basal hindlimb glucose oxidation rates did not differ among groups (Figure 3B). Insulin-stimulated glucose oxidation rates were lower (*p* < 0.05) for IUGR lambs but not IUGR+CLEN lambs than for controls. BUN concentrations were lower (*p* < 0.05) for IUGR lambs than for controls and were intermediate for IUGR+CLEN lambs, regardless of period (Figure 3C). Plasma HDL cholesterol concentrations were greater (*p* < 0.05) for IUGR and IUGR+CLEN lambs than for controls, regardless of period (Figure 3D). Basal plasma NEFA concentrations were lower (*p* < 0.05) for IUGR and IUGR+CLEN lambs compared to controls (Figure 3E). Plasma NEFA concentrations under HEC conditions were not different among experimental groups but were reduced (*p* < 0.05) from basal conditions for all groups. Plasma triglyceride concentrations were not different among groups but were lower (*p* < 0.05) during HEC conditions compared with basal conditions (Figure 3F). Blood K^+^ was lower (*p* < 0.05) and blood Ca^2+^ was greater (*p* < 0.05) for IUGR and IUGR+CLEN lambs than for controls, regardless of period (Figure 3G,H). Blood Na^+^ (Figure 3I) and Cl^−^ (109.5 ± 0.6 mM) were not different among groups or between study periods. Plasma insulin and blood glucose concentrations were lower (*p* < 0.05) for IUGR+CLEN lambs than for IUGR lambs and controls, regardless of period (Supplementary Figure S3A,B). Insulin-to-glucose ratios did not differ among groups but were greater (*p* < 0.05) by design during HEC than under basal conditions (Supplementary Figure S3C). Insulin sensitivity for glucose uptake tended to be greater (*p* = 0.07) for IUGR (1.91 ± 0.51 μmol/min/kg/μg/L) and IUGR+CLEN (2.25 ± 0.91 μmol/min/kg/μg/L) lambs than for controls (0.86 ± 0.17 μmol/min/kg/μg/L). Insulin sensitivity for glucose oxidation (0.36 ± 0.09 μmol/min/kg/μg/L) was not different among groups. Blood lactate concentrations (0.57 ± 0.03 mM) did not differ among groups or between study periods. Blood pH, hematocrit, and hemoglobin concentrations were lower (*p* < 0.05) for IUGR+CLEN lambs but not IUGR lambs than for controls, regardless of period (Supplementary Figure S4A–C). Base excess was greater (*p* < 0.05) for IUGR lambs but not for IUGR+CLEN lambs than for controls, regardless of period (Supplementary Figure S4D). Blood oxyhemoglobin was greater (*p* < 0.05) for IUGR lambs than for controls and was intermediate for IUGR+CLEN lambs (Supplementary Figure S4E). Blood HCO_3_^−^ (26.5 ± 0.3 mM) and pCO_2_ (38.6 ± 0.4 mmHg) did not differ among groups or between study periods. Blood pO_2_ was greater (*p* < 0.05) for IUGR lambs than for controls and was intermediate for IUGR+CLEN lambs (Supplementary Figure S4F). Carboxyhemoglobin concentrations were greater (*p* < 0.05) for IUGR and IUGR+CLEN lambs than for controls and greater (*p*< 0.05) for IUGR+CLEN lambs than IUGR lambs, regardless of period (Supplementary Figure S4G). Blood glucose, pCO_2_, Na^+^, and Ca^2+^ were greater (*p* < 0.05), and blood pH and oxyhemoglobin were lower (*p* < 0.05) for males than for females. Plasma insulin, blood glucose, base excess, and Ca^2+^ were lower (*p* < 0.05), and blood pH, HCO_3_^−^, oxyhemoglobin, and K^+^ were greater (*p* < 0.05) for twins than for singletons.

### 3.5. Whole-Body Oxidative Metabolism

**Figure 4:**
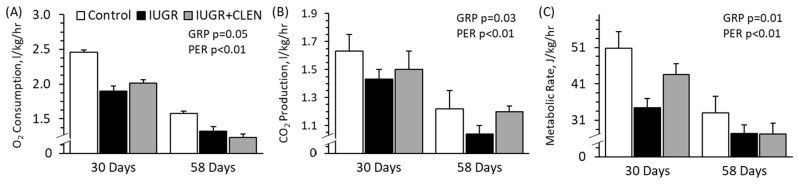
Whole-body oxidative metabolism in clenbuterol-treated IUGR-born lambs. Oximetry was performed at 30 and 58 days of age in controls (n = 13), untreated IUGR lambs (n = 11), and clenbuterol-treated IUGR lambs (IUGR+CLEN; n = 10). Data are presented for whole-body O_2_ consumption (**A**), CO_2_ production (**B**), and estimated metabolic (**C**) rates. Effects of the experimental group (GRP), age, and the interaction were evaluated and are noted where significant (*p* < 0.05).

No experimental group × age interactions were observed for any indirect calorimetry output. Whole-body O_2_ consumption rates were lower (*p* < 0.05) for IUGR and IUGR+CLEN lambs than for controls, regardless of age (Figure 4A). O_2_ consumption rates were also 36% lower (*p* < 0.05) at day 58 than at day 30 across all groups. Whole-body CO_2_ production rates were lower (*p* < 0.05) for IUGR lambs but not IUGR+CLEN lambs than for controls, regardless of age (Figure 4B). CO_2_ production rates were also 25% lower (*p* < 0.05) at day 58 than at day 30 across all groups. Metabolic rates were lower (*p* < 0.05) for IUGR lambs than for controls and were intermediate for IUGR+CLEN lambs, regardless of age (Figure 4C). Metabolic rates were also 32% lower (*p* < 0.05) at day 58 than at day 30 across all groups. CO_2_ production rates were lower (*p* < 0.05), and metabolic rates were greater (*p* < 0.05) for males than females. Metabolic rates were lower (*p* < 0.05) for twins than for singletons.

### 3.6. Ex Vivo Skeletal Muscle Glucose Metabolism

**Figure 5:**
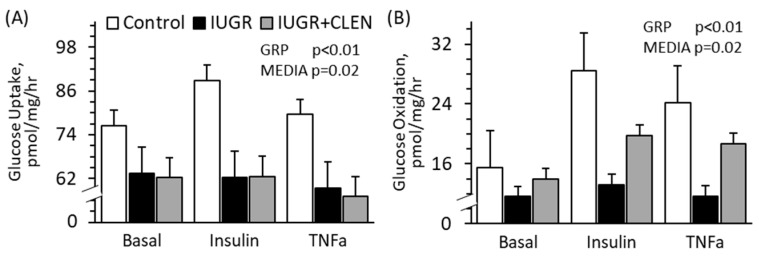
Ex vivo glucose metabolism in primary skeletal muscle from clenbuterol-treated IUGR-born lambs. Intact strips of flexor digitorum superficialis were isolated at 60 days of age in controls (n = 13), untreated IUGR lambs (n = 11), and clenbuterol-treated IUGR lambs (IUGR+CLEN; n = 10) and incubated with no additive (basal), 5 mU/mL insulin, or 10 ng/mL TNFα. Data are presented for glucose uptake (**A**) and oxidation (**B**) rates. Effects of the experimental group (GRP), media, and the interaction were evaluated and are noted where significant (*p* < 0.05).

No experimental group × media interactions were observed for ex vivo muscle glucose metabolism. Glucose uptake by FDS muscle was lower (*p* < 0.05) for IUGR and IUGR+CLEN lambs than for controls, regardless of media condition (Figure 5A). Glucose uptake was greater (*p* < 0.05) in insulin-spiked media than basal or TNFα-spiked media across all groups. Glucose oxidation by FDS was lower (*p* < 0.05) for IUGR lambs than for controls and was intermediate for IUGR+CLEN lambs, regardless of media condition (Figure 5B). Glucose oxidation was greater (*p* < 0.05) in insulin-spiked and TNFα-spiked media than in basal media across all groups. Glucose oxidation by FDS was also lower (*p* < 0.05) for twins than for singletons.

### 3.7. Muscle Protein and Glycogen Content

**Figure 6:**
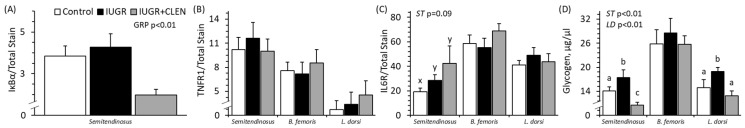
Skeletal muscle protein and glycogen content in clenbuterol-treated IUGR-born lambs. Semitendinosus, biceps femoris, and longissimus dorsi muscles were collected at 60 days of age from the controls (n = 13), untreated IUGR lambs (n = 11), and clenbuterol-treated IUGR lambs (IUGR+CLEN; n = 10). Data are presented for cellular IκBα (**A**), TNFR1 (**B**), IL6R (**C**), and glycogen (**D**) concentrations. Effects of the experimental group (GRP) were evaluated for each muscle and are noted where significant (*p* < 0.05). ^a,b,c^ Means with different superscripts differ (*p* < 0.05). ^x,y^ Means with different superscripts tend to differ (*p* < 0.10).

Representative gel images for Western immunoblots and MyHC electrophoresis are shown in Supplementary Figure S5A,B. The proportions of type I fibers estimated by MyHC-1 in *semitendinosus* muscle (37.8 ± 0.7%) were not different among experimental groups. The proportion of type IIa fibers estimated from MyHC-2a tended to be greater (*p* = 0.06) for IUGR+CLEN lambs (45.7 ± 1.7%) but not IUGR lambs (40.8 ± 1.2%) compared with controls (41.1 ± 0.8%). The proportion of type IIx fibers estimated from MyHC-2x was lower (*p* < 0.05) for IUGR+CLEN lambs (17.8 ± 0.7%) but not IUGR lambs (20.4 ± 1.1%) than for controls (20.8 ± 0.5%). *Semitendinosus* protein content for IκBα was lower (*p* < 0.05) for IUGR+CLEN lambs but not for IUGR lambs compared to controls (Figure 6A). *Semitendinosus*, *biceps femoris*, and *longissimus dorsi* protein content for TNFR1 was not different among groups (Figure 6B). *Semitendinosus* muscle protein content for IL6R tended to be greater (*p* = 0.09) for IUGR and IUGR+CLEN lambs than for controls (Figure 6C), but *biceps femoris* and *longissimus dorsi* protein content for IL6R did not differ among groups. *Semitendinosus* glycogen content was greater (*p* < 0.05) for IUGR lambs and lower (*p* < 0.05) for IUGR+CLEN lambs than for controls (Figure 6D). *Biceps femoris* glycogen content did not differ among groups. *Longissimus dorsi* glycogen content was greater (*p* < 0.05) for IUGR but not IUGR+CLEN lambs than for controls. *Biceps femoris* glycogen content and *semitendinosus* protein content for IL6R were lower (*p* < 0.05) for males than females. *Longissimus dorsi* glycogen content was greater (*p* < 0.05) and *semitendinosus* protein content for IκBα was lower (*p* < 0.05) for twins than for singletons.

### 3.8. Pancreatic Islet Morphology

**Figure 7:**
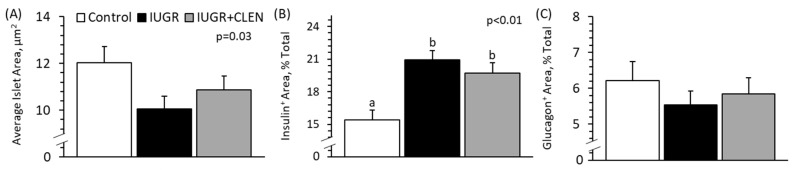
Pancreatic islet morphology in clenbuterol-treated IUGR-born lambs. Immunohistology was performed on pancreatic sections collected at 60 days of age from the controls (n = 13), untreated IUGR lambs (n = 11), and clenbuterol-treated IUGR lambs (IUGR+CLEN; n = 10). Data are presented for average islet area (**A**) and percentages of islet area staining positive for insulin (**B**) or glucagon (**C**). Effects of the experimental group were evaluated and are noted where significant (*p* < 0.05). ^a,b^ Means with different superscripts differ (*p* < 0.05).

Representative images of islet immunohistochemical staining are shown in Supplementary Figure S5C. The average pancreatic islet area was lower (*p* < 0.05) for IUGR lambs but not IUGR+CLEN lambs than for controls (Figure 7A). The percentage of insulin^+^ area in pancreatic islets was greater (*p* < 0.05) for IUGR and IUGR+CLEN lambs than for controls (Figure 7B). The percentage of glucagon^+^ area did not differ among groups (Figure 7C). The ratios of insulin^+^-to-glucagon^+^ area tended to be greater (*p* = 0.08) for IUGR lambs (7.32 ± 0.65) and IUGR+CLEN lambs (7.18 ± 0.79) than for controls (5.35 ± 0.78). The average islet area and percentage of insulin^+^ area were lower (*p* < 0.05), and the percentage of glucagon^+^ area was greater (*p* < 0.05) for twins than for singletons.

## 4. Discussion

In this study, we found that postnatal stimulation of β2 adrenergic activity reduced systemic inflammation and improved metabolic homeostasis in IUGR-born juvenile lambs. The loss of skeletal muscle β2 adrenoceptors and their downstream regulatory effects [13,14,17] following IUGR contributed to poor glucose oxidative capacity and greater glycogen content in muscle, as both were improved by postnatal clenbuterol injections. Elevated circulating NEFA concentrations in IUGR lambs were also ameliorated by the β2 agonist, consistent with its long-term inhibitory effects on lipolysis [33]. Although basal circulating insulin was normal, glucose-stimulated insulin secretion was reduced by almost half in IUGR lambs. The partial recovery of β cell stimulus–secretion coupling observed in clenbuterol-treated IUGR lambs was unexpected and may have been indirectly facilitated by the amelioration of elevated inflammatory cytokine secretion. Together, the improvements in indicators for systemic inflammation, muscle-centric metabolism, and β cell function observed when IUGR-born lambs were administered clenbuterol demonstrate that β2 adrenergic programming is a major underlying mechanism for poor metabolic homeostasis following fetal stress [34,35]. As such, this deviant β2 adrenergic regulation may represent an effective therapeutic target for improving metabolic outcomes following IUGR.

Diminished muscle-specific glucose oxidative capacity mediated by the loss of β2 adrenergic regulation reflects a persistent nutrient-sparing fetal programming mechanism in IUGR-born offspring. Skeletal muscle is the largest consumer of glucose [1,36], and even modest β2 adrenergic stimulation increases glucose oxidation rates in normal muscle and myoblasts [37,38]. However, desensitization of β2 adrenoceptors in IUGR muscle coincides with disruption of the TCA cycle and electron transport chain activity late in gestation [26,39,40]. This robust impairment of oxidative pathways manifests in less ATP production by the muscle tissues [3,26,39,40]. Programmed deficits in β2 adrenergic regulation of IUGR fetal muscle persist well after birth [14,17], and our present findings demonstrate their clear role in the hallmark metabolic dysfunction of IUGR offspring. In fact, 50% of the reductions in muscle-specific and consequent whole-body oxidative metabolic rates were recovered when IUGR lambs were treated daily with the β2 agonist. Interestingly, poor glucose oxidation was not concomitant with changes in muscle fiber type proportions. Moreover, it coincided with only small reductions in muscle glucose utilization that were not improved by β2 adrenergic stimulation. Conversely, the 25% greater glycogen accumulation in IUGR skeletal muscle was fully reversed by treatment with clenbuterol. In normal muscle, β2 adrenergic activity promotes glycogen mobilization [41] and slows its synthesis [42]. Even when β2 stimulation is brief, activated glycogen phosphorylase increases by up to 9-fold and glycogen synthase activity falls by 80% [43,44]. Following longer periods of stimulation, intramuscular glycogen content can be diminished by as much as 30% [45,46]. The suppressive effects of β2 adrenergic tone on systemic inflammation offer an additional mechanistic explanation for the observed metabolic phenotypes. Inflammatory cytokines disrupt insulin signaling, impair glucose metabolism in favor of lipid oxidation, and increase glycogen deposition [47]. In otherwise uncompromised fetuses, sustained inflammation in the near term causes deficits in muscle glucose metabolism even in the absence of other stress factors [4,27]. Our IUGR-born lambs exhibited substantially greater circulating TNFα and IL-6 concentrations that were completely ameliorated by daily clenbuterol administration. They also exhibited evidence of enhanced muscle sensitivity to inflammatory factors, albeit less robustly than observed in IUGR fetuses [27,36,48]. β2 agonists suppress leukocytic inflammasomes and reduce cytokine secretion into the bloodstream [49,50,51]. Reciprocally, experimental reduction of β2 adrenergic tone via pharmaceutical blockade or genetic knockout worsens inflammation [52]. In some studies, β2 agonists also suppressed intracellular inflammatory signaling pathways [53,54,55,56], although this was not apparent in the present study.

Metabolic dysfunction in IUGR-born lambs was accompanied by indicators of lipid dysregulation that were also responsive to β2 adrenergic stimulation. The observed normal blood triglycerides but elevated circulating NEFAs in IUGR-born lambs was comparable to lipid profiles reported in IUGR-born lambs, goats, and piglets [26,57,58]. Treating IUGR lambs with clenbuterol reversed the elevation in circulating NEFA, likely due to both direct and indirect effects on lipolysis. Short-term β2 adrenergic stimulation in adipocytes increases lipolytic rates and NEFA mobilization from fat deposits, although this response is dampened in IUGR-born offspring [12]. However, lipolytic responsiveness wanes after only a few days of sustained stimulation [59,60], and longer periods of stimulation ultimately produce anti-lipolytic effects [33]. Inflammatory cytokines also stimulate lipolysis [61], and thus the resolution of greater circulating TNFα and IL-6 in clenbuterol-treated IUGR lambs presumably further reduced circulating NEFA. Additionally, the anti-lipogenic effects of β2 pathways [62] may explain the concurrent increase of circulating triglycerides observed with clenbuterol, although this was independent of any IUGR effects. Regardless, hyperlipidemia, together with greater intramuscular fat content, may reflect a greater reliance on lipid utilization by IUGR muscle. A recent study in IUGR-born piglets found that elevated intramuscular lipid accumulation coincided with greater expression of fatty acid transporters [63]. Importantly, increased lipid utilization does not necessarily translate to increased fatty acid oxidation. A recent study in IUGR fetal sheep found that muscle-specific fatty acid oxidation was maintained as carbohydrate oxidation was diminished [64]. However, other studies in IUGR sheep, pigs, and rodents found that intramuscular lipid accumulation was coincident with elevated acylcarnitines, which indicate impaired fatty acid oxidation [65,66]. Although we did not directly assess fatty acid oxidation, our indicators for total oxidative metabolism indicate that deficits in glucose oxidation were not compensated by fat or protein oxidation in IUGR lambs.

Daily administration of clenbuterol to IUGR-born lambs improved poor glucose-stimulated insulin secretion despite only minimal influence on islet morphology. Circulating insulin concentrations were unperturbed by IUGR under resting conditions but exhibited a 40% disparity under hyperglycemia, demonstrating impaired stimulus–secretion coupling. Poor β cell function is a hallmark of IUGR fetuses late in gestation and is generally attributed to chronic fetal hypercatecholaminemia [18,19]. Infusion of catecholamines into uncompromised fetuses corroborates this by producing comparable suppression of insulin secretion [20]. However, impaired β cell function persists in IUGR-born neonates [14], despite hypercatecholaminemia being resolved at birth. Pancreatic islet function is profoundly regulated by α adrenergic pathways, with little influence from the β pathways [67]. Moreover, IUGR adaptations increase inhibitory α1 and α2 adrenoceptors in islets with no real impact on β receptor expression [18]. Despite this, postnatal clenbuterol recovered almost half of the deficit in glucose-stimulated insulin secretion observed in untreated IUGR lambs. We postulate that this effect was mediated by the suppression of circulating cytokines, which inhibit stimulus–secretion coupling in β cells by suppressing their glucose oxidation [68]. Impaired islet glucose oxidation is indeed the underlying mechanism for disruption of insulin secretion in several fetal IUGR models [69,70,71], and sustained inflammation by itself reduces glucose-stimulated insulin secretion in fetal sheep [4]. Islet dysfunction did not coincide with the obvious malformation of β cell populations. Although smaller in size, IUGR islets unexpectedly had 26% more insulin^+^ area (i.e., greater apparent β cell proportions) on average. The reason for this increase was unclear, as β cell populations are substantially diminished in IUGR fetal islets [30]. Moreover, compensatory expansion of β cell populations after birth was unlikely, as indicators of β cell proliferation remain reduced in IUGR offspring [72]. Postnatal clenbuterol treatment did not influence islet size or apparent β cell proportions. Although β2 adrenergic stimulation is critical for fetal islet development [73], the influence wanes soon after birth [74]. Regardless, changes in the observed morphological characteristics of islets did not appear to be requisite for improved β cell function in clenbuterol-treated IUGR lambs.

Metabolic improvements associated with clenbuterol treatment in IUGR lambs occurred without recovery of circulating IGF-1 concentrations. The majority of IGF-1 in the bloodstream originates from the liver, and IUGR conditions cause epigenetic modifications that reduce hepatic IGF-1 production [75,76]. This in turn reduces circulating concentrations by more than 60% in the fetus [77,78]. It is possible that local clenbuterol-induced changes occurred that were not reflected systemically, as long-term supplementation in rats increased skeletal muscle IGF-1 and IGF binding protein in skeletal muscle but actually reduced it in circulation [79].

## 5. Conclusions

From this study, we can conclude that IUGR-associated metabolic pathologies are caused in part by tissue-specific β2 adrenergic desensitization. Fetal programming of these adrenergic mechanisms was overcome in many ways by exogenous stimulation of β2 adrenergic activity with clenbuterol injections from the birth of the animal to the normal weaning age. Targeting reduced β2 adrenergic tone was particularly effective in recovering skeletal muscle glucose oxidation capacity, which coincided with improved whole-body oxidative metabolism. Impaired pancreatic β cell function that led to reduced glucose-stimulated insulin secretion did not appear to be associated with changes in β cell mass but rather due to impaired stimulus–secretion coupling. Moreover, increased β2 adrenergic activity improved the insulin secretion response to glucose. It is quite likely that much of the improvement associated with clenbuterol was indirectly facilitated by the suppression of systemic inflammation. However, not all indicators of metabolic health were improved by daily clenbuterol treatment, which illustrates the complexity of IUGR-associated pathologies and further indicates that multiple programming mechanisms contribute to postnatal metabolic deficits following heat stress-induced IUGR. Moreover, the less profound improvements in ex vivo metabolic assessments relative to in vivo assessments lead us to postulate that continuous treatment is required to mitigate the impact of reduced adrenergic tone in IUGR-born offspring.

## Data Availability

The datasets generated by this study will be made available upon reasonable request to the corresponding author. The data are not publicly available due to privacy.

## Supplementary Materials

The following supporting information can be downloaded at: https://www.mdpi.com/article/10.3390/metabo14030156/s1, Figure S1: Whole-body oximetry was assessed by indirect calorimetry using the Sable Systems Fox Box Respiratory System. Figure S2: Blood components in response to hyperglycemia in IUGR-born lambs supplemented with clenbuterol. Figure S3: Glucose homeostasis in response to hyperinsulinemia/euglycemia in IUGR-born lambs supplemented with clenbuterol. Figure S4: Blood components in response to hyperinsulinemia in IUGR-born lambs supplemented with clenbuterol. Figure S5. Representative micrographs for IUGR-born lambs supplemented with clenbuterol.
