# Supplementary material for: Daily Injection of the β2 Adrenergic Agonist Clenbuterol Improved Muscle Glucose Metabolism, Glucose-Stimulated Insulin Secretion, and Hyperlipidemia in Juvenile Lambs Following Heat-Stress-Induced Intrauterine Growth Restriction
Source: Metabolites. 2024 Mar 7;14(3):156. doi: 10.3390/metabo14030156 (PMC10972243; doi:10.3390/metabo14030156)
Supplement: Supplementary file 1 [file metabolites-14-00156-s001.zip › metabolites-2895748-supplementary.pdf]

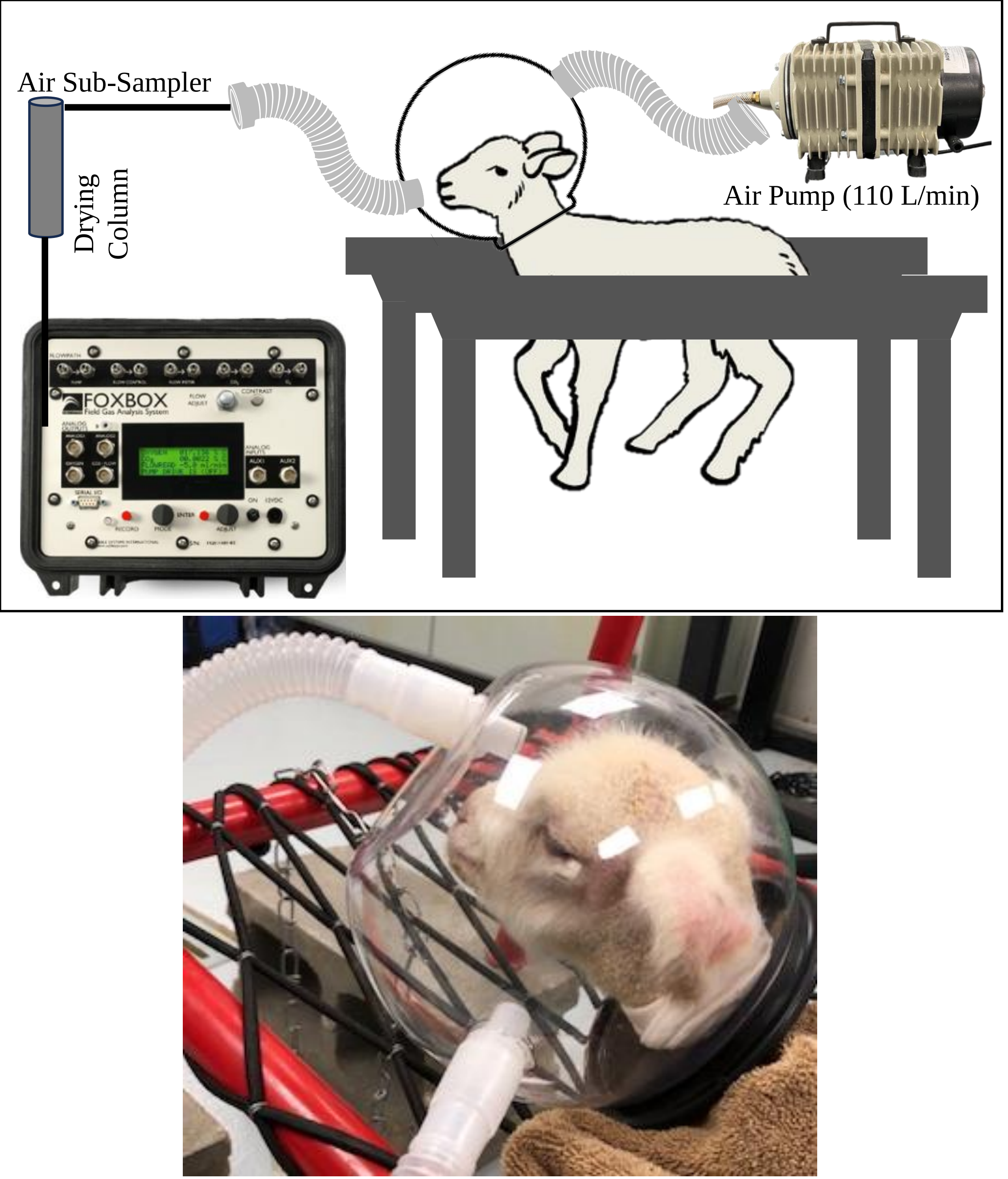

Supplementary Figure S1. Whole-body oximetry was assessed using Sable Systems Fox Box Respiratory System. A clear plastic globe was placed over the head and sealed to measure O_2 consumption and CO_2 production.

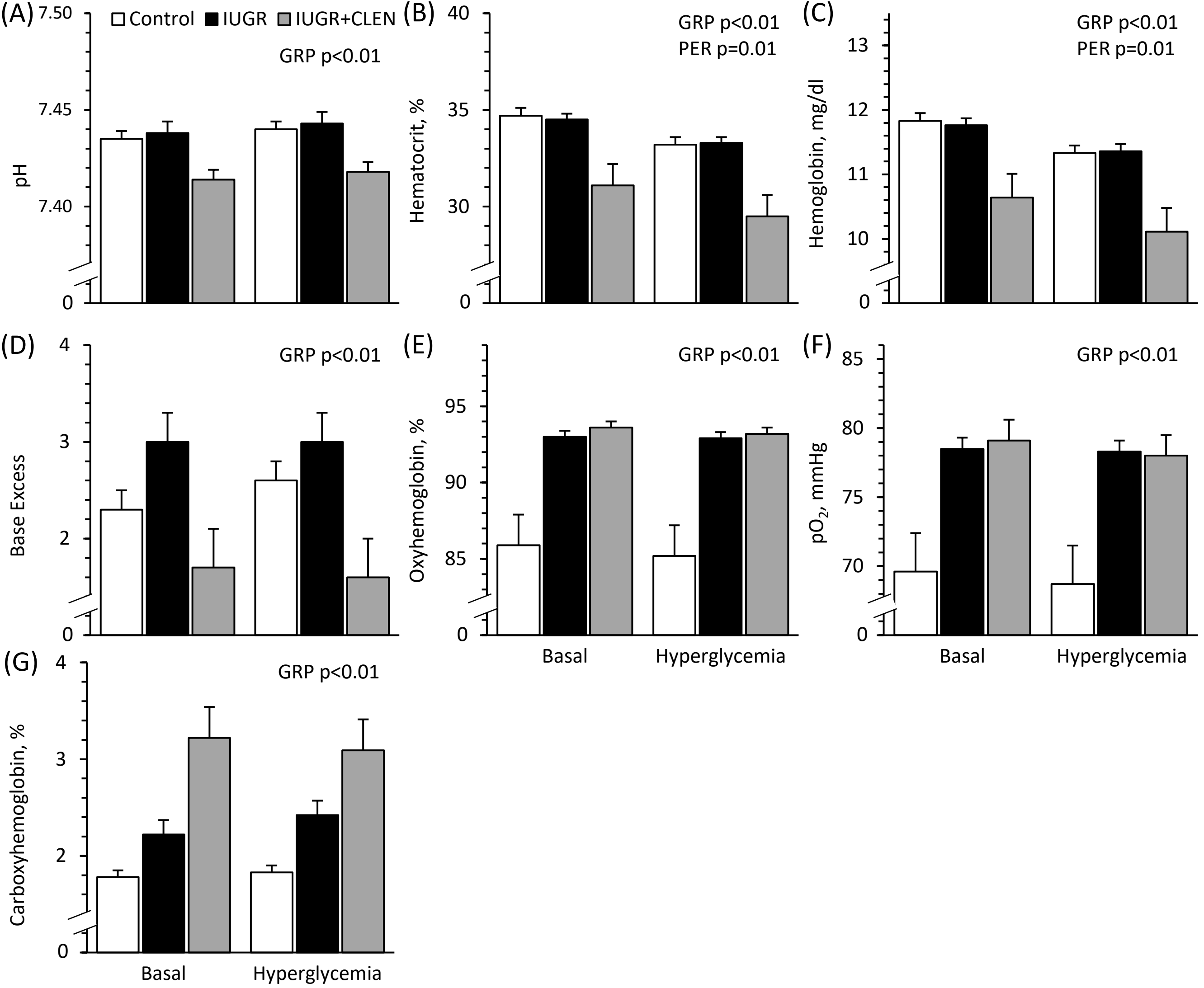

Supplementary Figure S2. Blood components during hyperglycemia in clenbuterol-treated IUGR-born lambs. Arterial blood samples were assessed under basal and steady-state hyperglycemia at 58 d of age in controls (n=13), untreated IUGR lambs (n=11), and clenbuterol-treated IUGR lambs (n=10). Data are presented for blood pH (**A**), hematocrit (**B**), hemoglobin (**C**), base excess (**D**), oxyhemoglobin (**E**), partial pressure of O_2 (**F**), carboxyhemoglobin (**G**), HCO_3 (**H**), and partial pressure of CO_2 (**I**). Effects of experimental group (GRP), period (PER), and the interaction were evaluated and noted where significant (p<0.05).

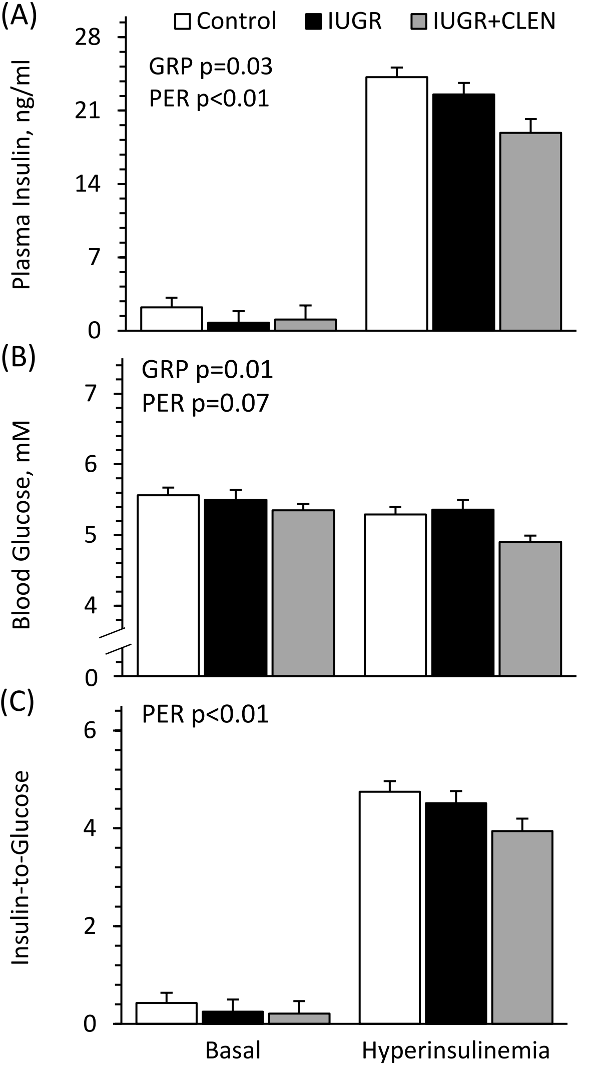

Supplementary Figure S3. Blood insulin during hyperinsulinemia in clenbuterol-treated IUGR-born lambs. Arterial blood samples were assessed under basal and steady-state hyperinsulinemia at 59 days of age in controls (n=11), untreated IUGR lambs (n=10), and clenbuterol-treated IUGR lambs (n=10). Data are presented for plasma insulin (**A**), blood glucose (**B**), and glucose-to-insulin (**C**). Effects of experimental group (GRP), period (PER), and the interaction were evaluated and noted where significant ($p<0.05$).

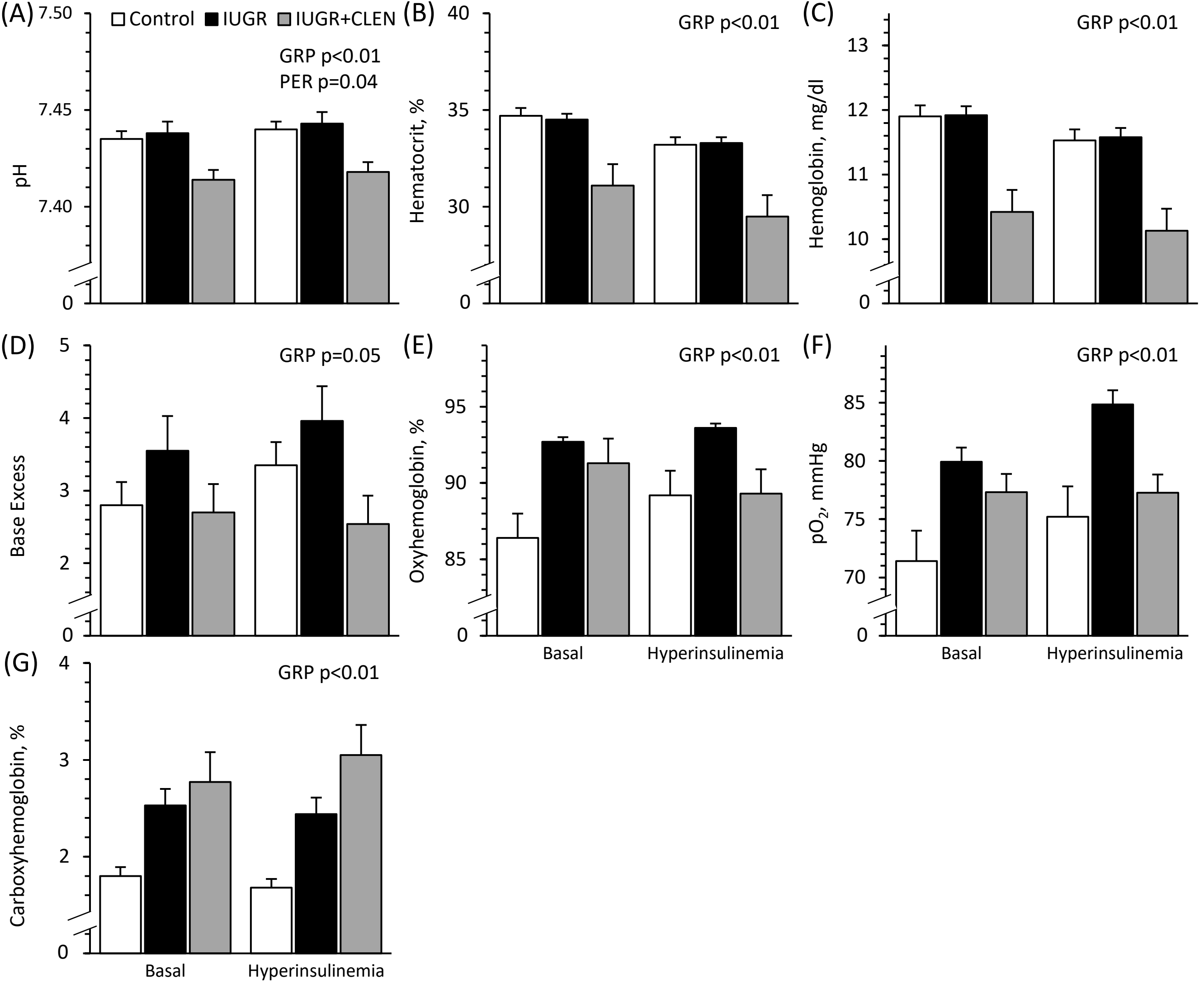

Supplementary Figure S4. Blood parameters during hyperinsulinemia in clenbuterol-treated IUGR-born lambs. Arterial blood samples were assessed under basal and steady-state hyperinsulinemia at 59 days of age in controls (n=11), untreated IUGR lambs (n=10), and clenbuterol-treated IUGR lambs (n=10). Data are presented for blood pH (**A**), hematocrit (**B**), hemoglobin (**C**), base excess **(D**), oxyhemoglobin (**E**), partial pressure of O_2 (**F**), and carboxyhemoglobin. Effects of experimental group (GRP), period (PER), and the interaction were evaluated and noted where significant (p<0.05).

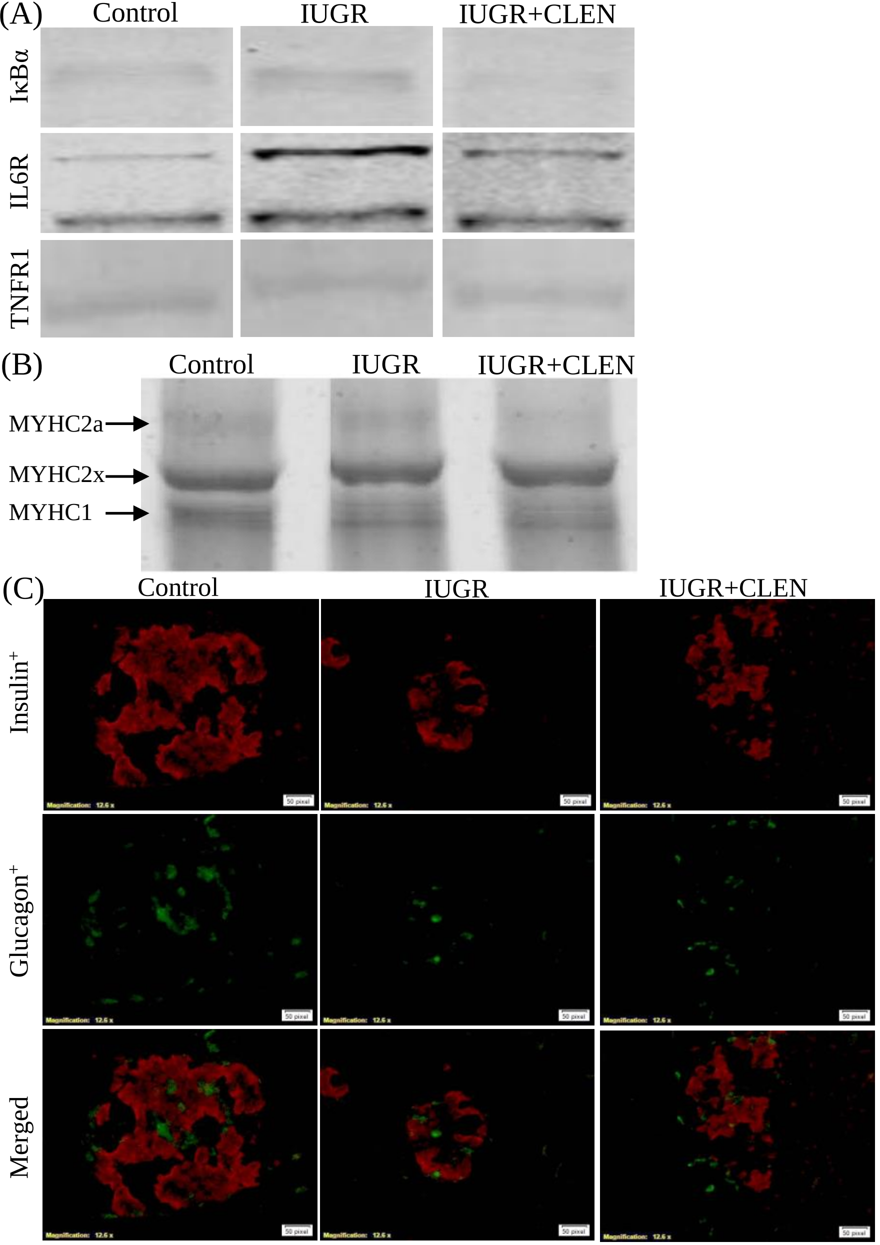

Supplementary Figure S5. Representative micrographs for IUGR-born lambs supplemented with clenbuterol. *Semitendinosus* muscle and pancreatic sections were collected at 60 days of age in controls (n=13), untreated IUGR lambs (n=11), and clenbuterol-treated IUGR lambs (n=10). Images are presented for muscle inflammatory protein westerns (**A**), muscle myosin heavy chain electrophoresis (**B**), and pancreatic islet staining (**C**).
